# Age-related promoter-switch regulates Runx1 expression in adult rat hearts

**DOI:** 10.1186/s12872-023-03583-3

**Published:** 2023-11-07

**Authors:** Jiawei Song, Xiaoling Zhang, Sinan Lv, Meng Liu, Xing Hua, Limin Yue, Si Wang, Weihong He

**Affiliations:** 1 Department of Physiology, West China School of Basic Medical Sciences and Forensic Medicine, Sichuan University, Chengdu, 610041 China; 2 Department of Cardiology, West China Hospital, Sichuan University, Chengdu, 610041 China; 3 Department of Neurology, West China Hospital, Sichuan University, Chengdu, 610041 China

**Keywords:** Runx1, Cardiovascular diseases, Cardiac ageing, Transcriptional regulation, DNA methylation

## Abstract

**Background:**

Runt-related transcription factor-1 (RUNX1), a key member of the core-binding factor family of transcription factors, has emerged as a novel therapeutic target for cardiovascular disease. There is an urgent need to fully understand the expression pattern of Runx1 in the heart and the mechanisms by which it is controlled under normal conditions and in response to disease. The expression of Runx1 is regulated at the transcriptional level by two promoters designated P1 and P2. Alternative usage of these two promoters creates differential mRNA transcripts diversified in distribution and translational potential. While the significance of P1/P2 promoter-switch in the transcriptional control of Runx1 has been highlighted in the embryogenic process, very little is known about the level of P1- and P2-specific transcripts in adult hearts, and the underlying mechanisms controlling the promoter-switch.

**Methods:**

To amplify P1/P2 specific sequences in the heart, we used two different sense primers complementary to either P1 or P2 5'-regions to monitor the expression of P1/P2 transcripts. DNA methylation levels were assessed at the Runx1 promoter regions. Rats were grouped by age.

**Results:**

The expression levels of both P1- and P2-derived Runx1 transcripts were decreased in older rats when compared with that in young adults, paralleled with an age-dependent decline in Runx1 protein level. Furthermore, older rats demonstrated a higher degree of DNA methylation at Runx1 promoter regions. Alternative promoter usage was observed in hearts with increased age, as reflected by altered P1:P2 mRNA ratio.

**Conclusion:**

Our data demonstrate that the expression of Runx1 in the heart is age-dependent and underscore the importance of gene methylation in the promoter-mediated transcriptional control of Runx1, thereby providing new insights to the role of epigenetic regulation in the heart.

**Supplementary Information:**

The online version contains supplementary material available at 10.1186/s12872-023-03583-3.

## Background

Heart disease is the leading cause of death on a global scale [1]. Many heart diseases, such as acute myocardial infarction, arrhythmias, hypertension, and valve disease, initiate a process referred to as cardiac remodelling, which leads to adverse changes in the architecture and function of the heart [2]. Progression of adverse cardiac remodelling is linked to the development of heart failure, resulting in increased deaths or hospitalizations [3]. Current medical therapy to prevent or reverse cardiac remodelling is inadequate, and therefore novel therapeutic strategies are needed to limit adverse remodelling and to improve prognosis in patients with heart disease [4]. Recently, runt-related transcription factor-1 (RUNX1) has been identified as a new therapeutic target that may achieve this objective [5]. RUNX1 is thought to be a master-regulatory transcription factor and has been most intensively studied in the hemopoietic system. While the function of Runx1 in the cardiovascular system has been discovered and Runx1 becomes a promising therapeutic target for protecting against adverse cardiac remodelling, the role that Runx1 plays in the heart merits further exploitation [2].

Runx1 is a member of the core-binding factor family of transcription factors implicated in multiple signaling pathways during normal development and disease [2]. Runx gene family encodes the α-subunits of a family of transcription factors that modulate proliferation, differentiation, and cell survival in diverse lineages. In mammalian species, three runt domain genes were identified, known as Runx1, Runx2, and Runx3 [6, 7]. All RUNX proteins bind to the same DNA motif and recruit common transcriptional modulators, but the spatial–temporal and tissue-specific expression patterns of each Runx gene are distinct on its own [7–11]. Runx1 is best characterized for its involvement in definitive hematopoiesis and hematological malignancies. Although the focus of Runx1 research has been predominately in the blood cancer field, recent evidence suggests that Runx1 has more widespread functions than previously considered. McCarroll & He et al. provide evidence for the first time that the up-regulation of Runx1 following myocardial infarction drives adverse cardiac remodelling [5]. At baseline, mice with Runx1-deficiency had equivalent contractile function to their littermate controls. However, following myocardial infarction notable differences in echocardiographic contractile parameters emerged with the progression of adverse cardiac remodelling. Whilst myocardial remodelling substantially occurred in control mice following myocardial infarction, characterized by declination of systolic function, thinning of the ventricular wall, and dilation of the ventricular chamber, the same parameters were absent in Runx1-deficient mice [5].

The expression of Runx1 is regulated at transcriptional level under the control of two promoters. In vertebrates, all Runx genes contain two alternative promoters, a distal P1 promoter and a proximal P2 promoter [12, 13]. The transcripts derived from P1 and P2 are differentially expressed in various cell types at different developmental stages [7, 14], and the dual promoter system is modulated in response to mitogenic stimulation in a cell type-specific manner [15, 16]. The alternative usage of P1 and P2 promoters produces mRNAs that differ in their 5'-regions including untranslated region (UTR) and N-terminal coding sequences, leading to the generation of protein isoforms [17, 18]. The P1- and P2-derived transcripts produce two major protein isoforms, RUNX1C and RUNX1B, respectively. These two isoforms differ in their N-terminal amino acid sequences, RUNX1C beginning with MASDS and RUNX1B beginning with MRIPV [17, 19]. The different sequences at the 5'-regions of Runx1 mRNA isoforms are important in the investigation of the promoter-specific transcription, because Runx1 mRNA transcripts derived from these two promoters can be monitored separately by using different sense primers complementary to either P1 or P2 5'-regions. This method is commonly used in the study of the hematopoietic system [14, 20–23]. The alternative usage of Runx1 promoters appears important for mediating tissue-specific and stage-specific Runx1 expression during hematopoiesis and development [14, 21].

The major protein isoforms produced by P1 and P2 are RUNX1C and RUNX1B, respectively, which differ in their N-terminal amino acid sequences. In the hematopoietic system, the function of Runx1 isoforms has been well studied [24]. RUNX1C is the most abundant isoform expressed in adult hematopoiesis and is tightly correlated with the expression of hematopoietic markers of differentiation [19]. RUNX1B is highly expressed in the megakaryocyte lineage but downregulated during erythropoiesis. RUNX1B participates in the process of hematopoiesis and negatively regulates the overgrowth of hematopoietic cells [25]. Recent studies have shown that RUNX1B is the earliest known marker of hematopoietic capacity in humans, and the appearance of RUNX1B in human endothelial cells is much earlier than previously reported hematopoietic markers, such as DLL4 and CD184. Chen et al. uncovered a direct relationship between RUNX1B expression and hematopoietic capacity, suggesting that RUNX1B expression in endothelial cells needs to reach a certain threshold to confer hematopoietic potential and to shift the balance from the endothelium to the primary state [24]. Consistently, the high expression of RUNX1B in newly generated hemangioblasts promotes hematopoietic capacity during mouse embryonic development [22, 26, 27]. Meanwhile, a key issue in early hematopoiesis is the development of mesodermal cells into a specific cell lineage that initially shares endothelial and hematopoietic potential. Draper et al. demonstrated that RUNX1B overexpression at an early stage strongly prevented the development of CD34 + endothelial and hematopoietic cells derived from mesodermal precursors [22]. These results suggest that Runx1B controls hematopoiesis at a moderate level and inhibits hematopoietic cell hyperplasia. Nevertheless, the functional roles of RUNX1C and RUNX1B protein isoforms in the heart have not been elucidated.

The transcriptional activity of Runx1 promoter is affected by gene methylation. Evidence from pluripotent murine embryonic stem cells shows that hypomethylation of P1 promoter is correlated with increased P1 transcriptional activity and increased P1:P2 mRNA ratio, suggesting an epigenetic regulatory mechanism underlying the Runx1 promoter switch and the resulting changes of Runx1 expression [28]. In the mammalian genome, gene methylation is an epigenetic mechanism contributes to the spatiotemporal regulation of gene transcription [29, 30]. Gene methylation occurs when a methyl group (CH3) is attached to DNA at its C-phosphate-G (CpG) site where a guanine (G) nucleotide is located next to a cytosine (C) and separated by only one phosphate (p) moiety in DNA sequence [31, 32]. Methylation of DNA leads to inhibition of transcription factors binding to DNA or recruitment of proteins related to gene repression, and thus plays a role in the transcriptional control of gene expression, which is dependent on the physiological condition and the properties of DNA itself. During development, the pattern of gene methylation changes via a dynamic process that entails both de novo DNA methylation and demethylation. As a result, differentiated cells such as cardiomyocytes and neurons have a more stable methylation pattern that enables tissue-specific gene regulation [30]. More recent advances show that gene methylation patterns are involved in chronic heart failure caused by dilated cardiomyopathy, and are associated with both normal homeostasis of cardiomyocytes and during cardiac stress, suggesting that gene methylation may play a role in the regulation of cardiomyocyte metabolism and contractile function [33–35]. It is unknown whether Runx1 expression in the heart is regulated by gene methylation.

In this study we have demonstrated that the expression of Runx1 in the heart, including P1- and P2-mediated transcripts as well as Runx1 protein levels, declined with increasing age in healthy adult rats, and in aged animals there was an alternative usage of Runx1 P1/P2 promoters observed. Furthermore, we have characterized the methylation pattern at two promoter regions of Runx1 gene and demonstrated that older rats had higher degrees of promoter methylation than younger adults. Taken together, these findings show that the alternative usage of Runx1 P1/ P2 promoters plays an active role in adult hearts during aging process and underscore the importance of gene methylation in the regulation of Runx1 expression.

## Results

### Runx1 promoter P1- and P2-mediated mRNA levels and Runx1 protein levels

**Fig. 1:**
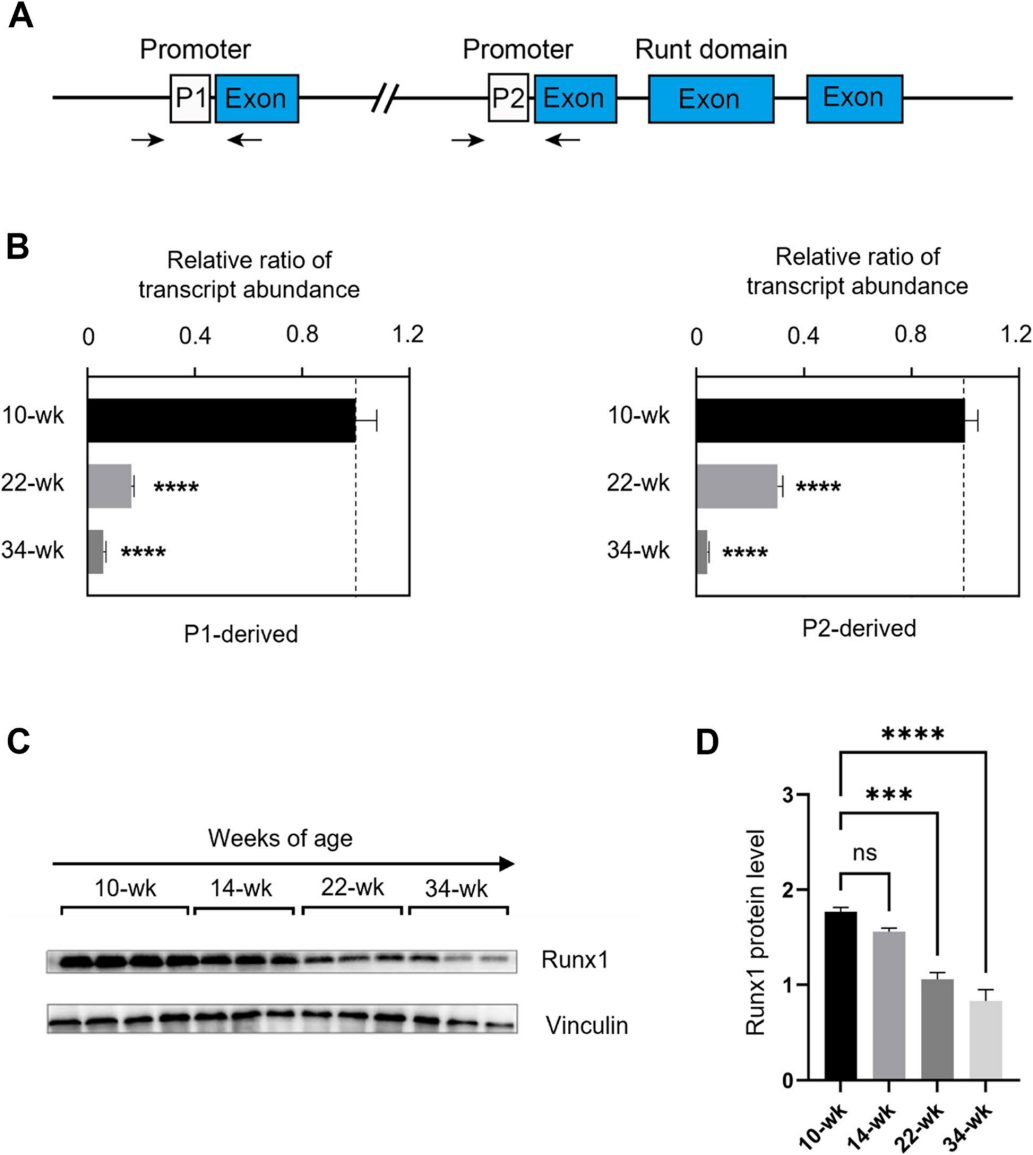
Runx1 promoter P1- and P2-mediated expression of Runx1 in rat hearts. **A** Schematic representation of Runx1 promoter region. Arrows mark positions of PCR primers used for detecting promoter-specific transcripts. **B** P1- and P2-mediated *Runx1* mRNA transcripts levels in whole heart homogenates from rats, expressed as ratios relative to the 10-wk level corrected for the corresponding β-actin abundance (mean ± SEM; *n* = 8 animals per group). ****P < 0.0001. **C** Representative western blot measuring levels of Runx1. **D** Densitometry quantification of Runx1 protein normalized using the related Vinculin band (mean ± SEM; *n* = 4 animals for 10-wk and *n* = 3 for other groups). ***P < 0.001 and ****P < 0.0001

Although Runx1 expression has previously been reported to increase following myocardial infarction and contribute to cardiac remodelling, it was unknown whether the baseline Runx1 expression in healthy animals changed with aging (eg. 34-week versus 10-week). Furthermore, the alternative usage of Runx1 promoters P1 and P2 (Fig. 1A) in adult hearts has not previously been investigated. Promoter-specific Runx1 mRNA expression was therefore quantified in hearts taken from rats aged 10, 22, and 34 weeks. Intriguingly, the levels of P1- and P2-mediated Runx1 mRNA both decreased with aging. P1-mediated Runx1 transcript demonstrated marked reductions in 22-week and 34-week hearts, which were 16% and 6% of the 10-week group, respectively. Similar reductions were also observed in P2-mediated transcript. The P2-derived mRNA levels at 22-week and 34-week were 30% and 4% of the 10-week group, respectively (Fig. 1B). To corroborate that the decreased expressions of Runx1 mRNA were consistent with decreased protein expression, we analyzed Runx1 protein levels (52 kDa isoform) within 10-, 14-, 22-, and 34-week rats. The levels of Runx1 protein changed in line with mRNA level. Runx1 protein levels decreased to 88%, 59%, and 47% in 14-, 22-, and 34-week hearts, respectively, relative to the protein level at the 10-week time point (Fig. 1C and D), suggesting a progressive decline of Runx1 expression with increasing age.

### DNA methylation of Runx1 promoter P1 and P2 regions

**Fig. 2:**
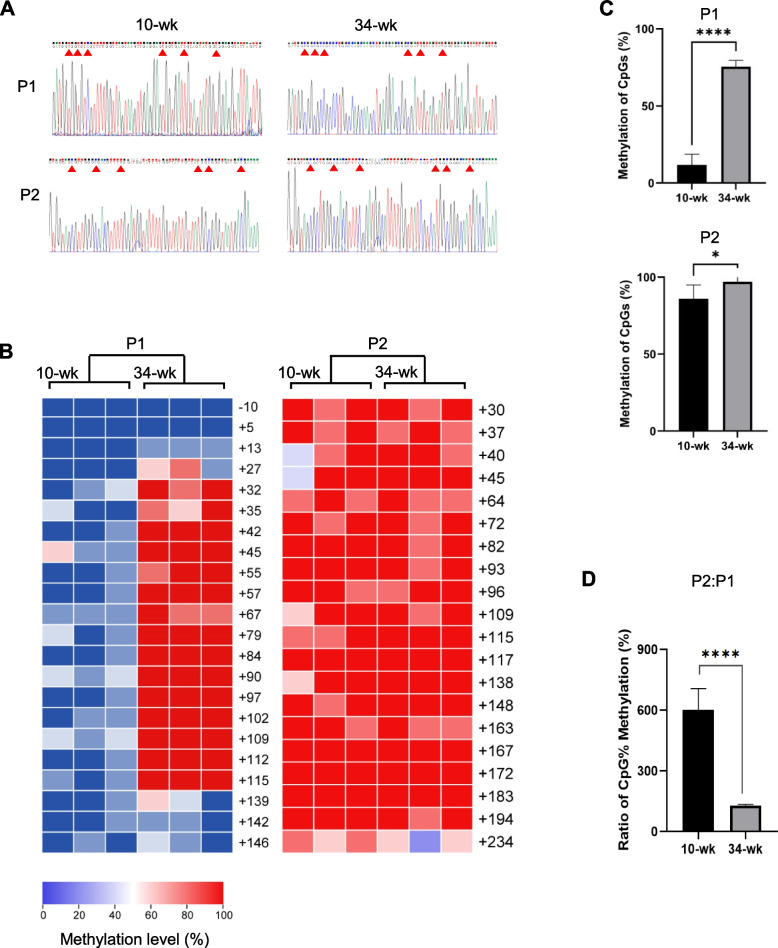
Bisulfite sequencing analysis of Runx1 P1 and P2 promoter regions. **A** Examples of sequencing readouts after bisulfite treatment. Red arrows indicate CpG sites where unmethylated cytosine was converted to thymine after PCR amplification and methylated site remained unconverted. **B** Heatmaps of representative CpG sites examined. Methylation levels were displayed as colors ranging from blue to red as shown in the key. **C** Quantification of percent methylation of CpG sites at Runx1 P1 and P2 promoter in 10- and 34-week rats, and (**D**) ratio between P2 and P1 region (mean ± SEM; *n* = 15 samples, 3 hearts). *P < 0.05 and ****P < 0.0001

We next investigated whether DNA methylation patterns at Runx1 promoter regions altered with aging (because DNA methylation can reduce gene expression). Bisulfite sequencing analysis (Fig. 2A) of Runx1 P1 and P2 promoter regions shown that the methylation levels at the P1 and P2 promoter regions in 34-week hearts increased by 6.4-fold and 1.1-fold, respectively, relative to the 10-week rats (Fig. 2B and C), indicating that gain of methylation at Runx1 promoter regions was correlated with decreased Runx1 expression. To further define the differential methylation patterns of P1 and P2 promoters, we compared the methylation level of P2 promoter to that of P1 and found an increase of 7.3-fold and 1.3-fold in 10- and 34-week hearts, respectively, with the former methylated to a higher degree (Fig. 2B and D). It is interesting to note that although the methylation level of P2 was greater than that of P1, the P2:P1 methylation ratio dramatically reduced in 34-week hearts compared to 10-week hearts (Fig. 2D), suggesting an alternation of P1 and P2 promoter usage (affected by DNA methylation) occurred as rats growing older.

### Switch of Runx1 promoter usage when hearts getting older

**Fig. 3:**
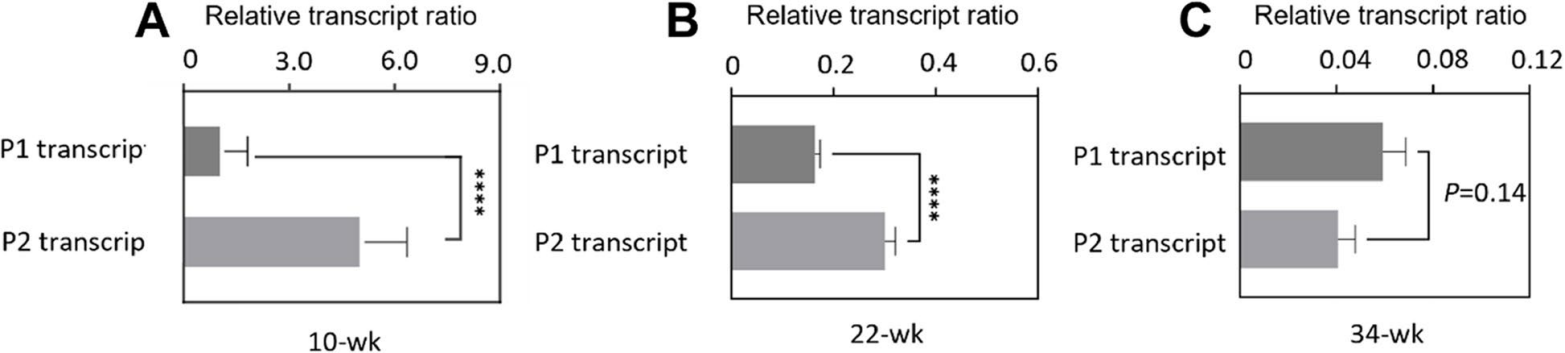
Alternative Runx1 P1 and P2 promoter usage in the process of aging. P1- and P2-mediated Runx1 mRNA transcripts levels in whole heart homogenates from (**A**) 10-wk (**B**) 22-wk (**C**) 34-wk rats. Transcripts levels at 22-wk and 34-wk were expressed as ratios relative to the 10-wk level corrected for the corresponding β-actin abundance (mean ± SEM; *n* = 8 animals per group). **** P < 0.0001

To investigate the alternative usage of two Runx1 promoters during the process of aging, we examined the mRNA levels of P1- and P2-mediated transcripts in 10-, 22- and 34-week hearts. The results shown the course of transcriptional alteration from age 10-week to age 34-week (Fig. 3A, B and C). Whilst the abundance of P2 mRNA transcript was 184% of that of P1 mRNA transcript in 22-week rats, no statistically significant differences were detected in 34-week rats (Fig. 3B and C). These observations indicate that P2 promoter had a greater activity compared to P1 in younger rats (aged 22 weeks) and may constitute the majority of Runx1 expression at this stage. However, when rats grew older to 34 weeks, the contribution of P1 promoter increased to some extent, and the P1:P2 mRNA ratio increased from 0.6 at 22-week to 1.7 at 34-week. This phenomenon of alternative promoter usage is referred to as promoter-switch which has previously been observed during embryogenesis and it was thought to play a role in mediating tissue and stage specific expression of Runx1 during embryonic development [14].

## Discussion

Recent experimental studies on the role of Runx1 in the adverse cardiac remodelling following myocardial infarction have attracted substantial attention of the cardiovascular field [2, 5]. Since Runx1 has been identified as a new therapeutic target for developing drugs to treat cardiovascular diseases, it is important to get a better understanding of its expression patterns and regulatory mechanisms. In this study, we characterized the expression of Runx1 in rat hearts of different age groups. Specifically, we quantified the transcripts of Runx1 mediated by 2 different promoters and examined the methylation patterns at the corresponding promoter regions. These results suggest that expression of Runx1 can be modulated by gene methylation in adult rat hearts.

Our results provide the first quantification of Runx1 expression in adult rat hearts with different age groups. Using 10-, 22- and 34-week-old rats, we demonstrated an age-dependent decrease in Runx1 mRNA levels derived from both P1 and P2 promoters (Fig. 1B-D). A similar age-dependent decrease was also observed in the Runx1 protein levels. One possible explanation for the observed changes of Runx1 expression is the reduction of heart’s endogenous capacity for regeneration with increasing age [36], given that Runx1 is thought to play a contributing and non-hemogenic role in the heart during embryonic development [2]. The importance of Runx1 in heart development is highlighted in Runx1 knockout mice. These mice show an underdeveloped coronary plexus, smaller ventricular free wall vessels and changes in heart structure, such as ventricular septal defects and thin myocardium [37]. Significantly, expression of Runx1 in neonatal hearts is higher compared with adult hearts [38]. This coincides with our observation that Runx1 expression is higher in young adults than in old ones, suggesting the declining trend of Runx1 expression in the heart continues in adulthood, although we speculate that the rate of declination in adulthood might be slower than in neonatal stage.

Although decreased expression of Runx1 was observed in the adult heart, previous studies have demonstrated that Runx1 expression is increased in response to cardiac injury [5, 39–41]. McCarroll & He et al. using mice with an inducible cardiomyocyte-specific Runx1 deficiency demonstrated that the activation of Runx1 following myocardial infarction is not only a marker of cardiac damage but also plays a role in the progression of adverse ventricular remodeling [5]. The effect of Runx1-deficiency on cardiac structure and function of mouse hearts can be explained by improvements in calcium homeostasis in cardiomyocytes. At 2 weeks following myocardial infarction, cardiomyocytes isolated from Runx1-deficient hearts had an increased amplitude of sarcoplasmic reticulum (SR)-mediated Ca^2+^ release stimulated electrically, a faster removal rate of calcium from the cytosol by the SR Ca^2+^-ATPase (SERCA) and a higher SR content [5]. The protection afforded by Runx1-deficiency is in line with other studies that shown improvements in cardiomyocyte calcium handling contributed to a notable beneficial effect on function and myocardial architecture [42–44]. In the present study, we have established a baseline knowledge regarding the steady-state expression of Runx1 in aged rats. This baseline knowledge is useful for the following factors: (1) whilst most of the laboratory animal studies performed to date use young adults, older people are more likely than the young to suffer from heart diseases [45]; (2) aging leads to a progressive decline in cardiac structure and function [46]; and (3) cardiac dysfunction caused by insults may not be apparent in young adults but may become manifest in old ones. This age-related cardiac dysfunction was highlighted by Khan et al. and Chen et al. using experimental mouse models involving polymicrobial sepsis-induced cardiac dysfunction [47, 48]. In the aged model of sepsis-induced cardiac dysfunction, mice aged 2, 5, and 8 months old demonstrated an age-dependent decrease in systolic contractile function [47]. Given that the baseline expression of Runx1 is comparatively low in the old but healthy animals as we report here, its functional role might not be prominent under normal conditions. Nevertheless, the expression of Runx1 can be activated in response to injury, and then the activated Runx1 expression can lead to decreased contractility and cardiac dysfunction [5]. Altogether our data implicate that the activation of Runx1 might be a contributor to age-related cardiac disorders, although the precise mechanisms of how Runx1 expression is triggered and how it affects cardiac function are not fully understood.

To investigate the specific contribution of Runx1 P1/P2 promoters in the heart to the modulation of Runx1 expression, we used two different sense primers complementary to either P1 or P2 5'-regions to monitor the promoter specific expression (Fig. 1A). It is well documented that the expression of Runx1 is mediated by 2 mutually distinct promoters P1 and P2, and that the alternative usage of these promoters allows diversified transcriptional control [14, 20, 21, 49]. Here we report that the expression levels of both P1- and P2-derived transcripts were decreased in older rats (22 and 34 weeks) when compared with young rats (10 weeks), consistent with the progressively decreased Runx1 protein levels observed (Fig. 1B-D). Furthermore, an alternative promoter usage was found in aged hearts as reflected by altered P1:P2 mRNA ratio (Fig. 3A and B). This phenomenon is referred to as promoter-switch which is also observed in embryonic development and hematopoiesis [14]. During mice embryogenesis, the alternative usage of Runx1 promoters regulates the spatiotemporal expression of Runx1 developmentally by giving rise to transcriptional variants and structurally different protein isoforms [14]. In adult hematopoiesis, whilst the P1 promoter is broadly active in hematopoietic stem and progenitor cell populations, the activity of the P2 promoter is comparatively restricted and its upregulation coincides with a loss of erythroid lineage specification [22].

As a key transcriptional regulator, RUNX1 interacts with a large number of transcription factors and cofactors, including P300, E2A-PBX1, Src-1. P300 is a kind of the histone acetyltransferases, which induces histone H3 lysine 27 acetylation (H3K27ac) at target gene promoters, enhancers and super-enhancers [50]. Yamaguchi et al. revealed that p300 acetylates Runx1 at the two conserved lysine residues. Runx1 is subject to acetylation at the same sites in vivo, and p300-mediated acetylation significantly augments the DNA binding activity of Runx1 [51]. In the adult heart, p300 is differentially expressed in different regions, and it has been suggested that p300 is a possible target for ameliorating aging-related cardiovascular diseases [52]. The amplitude of the P300 increases from childhood to early adulthood, whereas from early adulthood to old age the P300 amplitudes progressively decrease [53, 54]. E2A is a transcription factor that binds to DNA and interacts with other transcription factors and regulators to control the expression of specific genes [55, 56]. E2A-PBX1 fusion can lead to changes in gene expression that promote cell proliferation and differentiation, which are often associated with the development of acute lymphoblastic leukemia (ALL) [57]. Runx1 can activate E2A-PBX1 expression and Runx1 itself can also be activated by E2A-PBX1 as observed in E2A-PBX1 + leukemia [58]. The expression of E2A-PBX1 is age-related and progressively decreased with aging in senescent B cells. Src-1 is a steroid receptor coactivator that activates the transcription of Runx1 [59]. Src-1 is required for cardiomyocyte proliferation and differentiation during early developmental stages, and its dysfunction leads to abnormalities in neonatal and adult mouse hearts [60]. Immunochemical analysis shown Src-1 expression in proliferating cardiomyocytes of mouse hearts during the prenatal and neonatal stages, whereas its expression disappeared within the first two weeks after birth [61]. In addition, Src-1 mRNA and protein levels were found to decrease with aging [60]. Decreased SRC-1 expression was also observed in the brain of Japanese quail and spinal cord of rats after adulthood [62, 63].

It is noteworthy that genes with RUNX1-binding sites at their promoter region are overrepresented in a group of genes that become methylated in early life [64]. Since increased gene methylation switches off genes necessary for heart development and supports transition to a more adult phenotype, this process may be important for maturation. Particularly, methylation of Runx1 target genes during the first week after birth coincides with decreased capacities of hearts proliferation and regeneration [64]. Given that Runx1 expression is reduced after birth as mentioned above, it is conceivable that Runx1 itself may also be methylated together with its target genes. Here we report that the methylation levels at Runx1 promoter regions in aged hearts were higher than in young hearts (Fig. 2B and C), and the increased methylation was paralleled with decreased Runx1 expression. This implies a negative correlation that exists between Runx1 promoter methylation and gene expression and is supported by the well-known mechanism that promoter methylation usually leads to gene repression by blocking the formation of transcriptional complex [65]. Nonetheless, it is notable that methylation of gene may not necessarily lead to silencing. When comparing a collection of specific gene sites across multiple samples, several studies show the opposite trend: gene hypermethylation can be associated with increased expression of nearby genes [66–69]. In addition, variations of methylation level was observed in cancer cells, and it was argued that the aberrant hyper- or hypo-methylation was correlated with activation of oncogenes and/or repression of tumor suppressor genes [70]. Indeed, DNA methylation at regions near promoter is more likely to be associated with transcriptional repression, and this inversed correlation is supported by several studies investigating promoter methylation [71–76].

The present study shown that although the degree of methylation at P2 region was higher than that of P1, the P1:P2 methylation ratio dramatically increased in aged rats compared with young rats (Fig. 2D). The change of methylation ratio in aged hearts is consistent with increased P1: P2 mRNA ratio. These observations suggest that the alternative usage of Runx1 promoter may be caused by promoter methylation which can lead to gene silencing. The changed P1:P2 methylation ratio might be related to the specific location of CpG islands (regions of the genome which contain a large number of CpG repeats). There are two CpG islands near the P2 promoter, but none near the P1 promoter [77]. Several studies shown that the impact of DNA methylation on gene expression is dependent on whether the methylation is located in or near the CpG islands [67, 68]. Whereas methylation of CpG islands at promoter region tends to generate stable silencing of associated genes [30, 78], our data shown that P1 promoter (without a CpG island located nearby) appeared to be more unstable than its counterpart P2, with greater fluctuations observed in both methylation and transcription levels (Fig. 2D and Fig. 3). This speculation about methylation stability is in line with a more recent study carried out in hematopoietic cells where the methylation status of P1 underwent a drastically change over hematopoietic differentiation while P2 region remained comparatively stable [79]. Another explanation for the methylation change is aging. There is thought to be a correlation between epigenetic changes of DNA and the biological age of organisms and tissues. Based on this correlation, epigenetic researchers proposed a method to predict both chronological and biological age, a method which involves measuring changes in DNA methylation, specifically the methylation status of certain CpG sites [80–84]. Alongside age prediction, these studies proved that some of the CpG sites on the genome can have age-related methylation changes. Similarly, our results demonstrated that the methylation pattern of CpG sites within Runx1 promoter regions altered with increasing age. Detailed mechanisms underlying these age-related changes warrant further study.

The limitation of this study is that it did not include functional analysis to confirm the role of differential methylation but provided only correlative evidence. In the previous study, McCarroll & He et al. have performed functional analysis following myocardial infarction and shown that the up-regulation of Runx1 causes decreased cardiac function [5], whereas the mechanisms underlying the regulation of Runx1 expression are unclarified. Therefore, the present study aimed to observe the expression pattern of Runx1 and explore the potential mechanisms contributing to the regulation of Runx1 expression. Here we shown that increased methylation was paralleled with decreased transcriptional activities of Runx1 promoters, implying that DNA methylation is responsible for the change of transcriptional activities. It is evidenced that in mESC-derived hematopoietic cells Runx1 P1 promoter interacts with transcription factor HOXB4 by the maintenance of methyltransferase DNMT1. Decreased methylation and acquisition of active chromatin modifications lead to increased transcriptional activity of the P1 promoter in vitro via the physical interaction with HOXB4 [28, 85, 86]. These results together with our data suggest that different methylation patterns are associated with different transcriptional activities of Runx1 promoters.

## Conclusion

We have demonstrated for the first time that P1- and P2-mediated Runx1 transcripts decrease with increasing age and highlighted the importance of gene methylation in the transcriptional control of Runx1 expression. Previous studies established that Runx1 overexpression drives cardiac dysfunction by affecting calcium homeostasis in cardiomyocytes after myocardial infarction [2, 5]. A more recent study by He et al. demonstrated similar deteriorations of calcium handling in the context of myocardial ischemia/reperfusion injury [87]. As a newly identified target of interest [2], Runx1 can be further exploited in basic and translational studies to protect against myocardial ischemia/reperfusion injury and/or to limit the progression of adverse cardiac remodeling, thereby improving therapeutic strategies and patient prognosis of heart diseases.

## Methods

### Animal husbandry

Male Wistar rats were obtained from Chengdu Dashuo Experimental Animal Co. Ltd, and housed in standard environmental conditions (at 20–22 °C with 12 h light–dark cycle). Rats were fed with standard food (Formula Diet for rodents; Dashuo, Chengdu, China) and high purity water. All animal procedures were approved by the Sichuan University Medical Ethics Committee and the study was conducted in accordance with the ARRIVE guidelines. The care and use of animals were in accordance with local and international ethical criteria.

### Blind method

The experimental operators themselves did not know the grouping of the experiment, and animals were uniformly assigned by the laboratory staff.

### Treatment of randomization

The experimental animals were numbered and divided into experimental group or control group by random functions.

### Criteria to exclude experimental animal

Health: Experimental animals must be in good health and free of any disease or viral infection. Any animal showing unusual symptoms, such as dyspnea, diarrhea, etc. was excluded from the experiment.

Age and Sex: Animals of same age and sex were used to reduce error.

### Runx1 promoter-specific RT-PCR

The animals were anesthetized in an induction chamber with 4% isoflurane and 100% oxygen. The anesthetized rats were sacrificed by cervical dislocation and the heart was removed and washed in ice-cold saline (0.9% sodium chloride w/v). Total RNA was extracted by Trizol reagent(Invitrogen, USA) following the manufacturer’s guidelines. cDNA was isolated by Extraction of FastKing one-step RT-PCR kit (Tiangen, China). Real-Time PCR was performed by SYBR Green probes (Bio-Rad, USA). Two different sense primers complementary to either P1 or P2 5'-regions were used to amplify P1/P2 specific sequences. β-actin was used as an internal control. Relative quantification was calculated using the 2^−ΔΔCt^ method after the threshold cycle. The primer sequences used for PCR were list in Table 1.

**Table 1:** Primers used for assessing transcript abundance and DNA methylation

Primer’s assay	Target	Primer Sequence 5`-3`direction	Product Size
qPCR	P1 mRNA F	GAAGTGTAAGCCCAGCACAGT	103
	P1 mRNA R	GGCGGGGGATTCGATGATTT	103
	P2 mRNA F	AAGATCCGAGTCCCTGTC	70
	P2 mRNA R	TCACAACAAGCCGATTGAGT	70
	β-actin F	GATGACGATATCGCTGCGCTG	201
	β-actin R	GTACGACCAGAGGCATACAGG	201
Bisulfite sequencing	P1 outer primer F	TAGTAGGAGTTTTTAGGGTTTT	400
	P1 outer primer R	GTATGGTGGAGGTATTAGTTGATTATT	400
	P1 inner primer F	AATAATCAACTAATACCTCCACCATAC	213
	P1 inner primer R	CAACTAATACCTCCACCATACTAC	213
	P2 outer primer F	GTTTTTTTTTGGTTGGTGTTTAGG	260
	P2 outer primer R	ATAACCTCAATAACAAAAAACCACTACT	260
	P2 inner primer F	GTTTTTTTGGTTGGTGTTTAGGTGGTAG	253
	P2 inner primer R	CCTCAATAACAAAAAACCGCTACTAACTAC	253

*Abbreviations*: *F* forward primer, *R* reverse primer

### Western blotting

Total protein samples were extracted from rat heart tissues mixed with ice-cold RIPA protein lysis buffer (Beyotime, China). Protein concentrations were detected by the BCA kit (Beyotime, China). Protein extracts with loading dye were loaded into SDS-PAGE gels. The extracts (30 μg protein) were planished in the lane of 5% gels and separated with 12% SDS-PAGE gels. Bands were transferred into a PVDF membrane, blocked with 5% skim milk at room temperature for 1 h, and dipped into rabbit anti-Runx1 antibodies (1:1500 dilution;ab92336, USA) and rabbit anti-Vinculin antibodies (1:3000 dilution, A2752,abclone, China) overnight at 4 °C followed by 1 h treatment of secondary antibody HRP-labelled goat anti-rabbit IgG (1:5000 dilution, AS014, abclone, China) at room temperature. ECL Chemiluminescence substrate (Biosharp, China) was used to visualize the blots. Band densities were quantified by ImageJ software.

### Bisulfite sequencing

DNA of rat hearts was extracted using Cell/Tissue DNA Kit (GBC, China). The genomic DNA was converted by using sodium bisulfite (Tiangen, China). After sodium bisulfite conversion, unmethylated cytosines were converted to uracil, whereas methylated cytosines unconverted. Primers to amplify converted genomic DNA were designed by Methprimer (http://www.urogene.org/methprimer/index1.html). Nest-PCR amplification with outer and inner primer pairs was used in order that target sequences can be amplified abundantly. PCR mix (Tsingke, China) was used for amplification. Amplification products were visualized by gel electrophoresis and bands were cut and purified using Generic Agarose Gel DNA Recovery Kit (Tiangen, China). Pure PCR products were inserted into the pClone007 Simple Vector Kit (Tsingke, China) and individual clones were sequenced. Alignment and methylation analyses were performed by using QUMA (http://quma.cdb.riken.jp/). The primer sequences used for PCR were list in Table 1.

### Statistics

Data were analyzed using GraphPad Prism software and are presented as mean and SEM. For statistical analysis of differences between groups, an unpaired t test (2 groups), or a 1-way ANOVA (more than 2 groups) were used as appropriate. Dunnett’s posttest was used for multiple comparison. Values of *P* < 0.05 were considered to be statistically significant. Outliers were excluded to prevent distorting the overall data.

### Supplementary Information

**Additional file 1.**

**Additional file 2: Supplemental Material Figure 1.** Original Western blot images for Fig. 1C.

## Data Availability

The datasets used and/or analysed during the current study are available from the corresponding author on reasonable request.
