# Supplementary material for: Age-related promoter-switch regulates Runx1 expression in adult rat hearts
Source: BMC Cardiovasc Disord. 2023 Nov 7;23:541. doi: 10.1186/s12872-023-03583-3 (PMC10631011; doi:10.1186/s12872-023-03583-3)
Supplement: Supplementary file 2 — Additional file 2: Supplemental Material Figure 1. Original Western blot images for Fig. 1C. [file 12872_2023_3583_MOESM2_ESM.pdf]

Age-related promoter-switch regulates Runx1 expression in adult rat hearts

Jiawei Song, Xiaoling Zhang, Sinan Lv, Meng Liu, Xing Hua, Limin Yue, Si Wang and Weihong He

Supplemental Material Figure 1

a

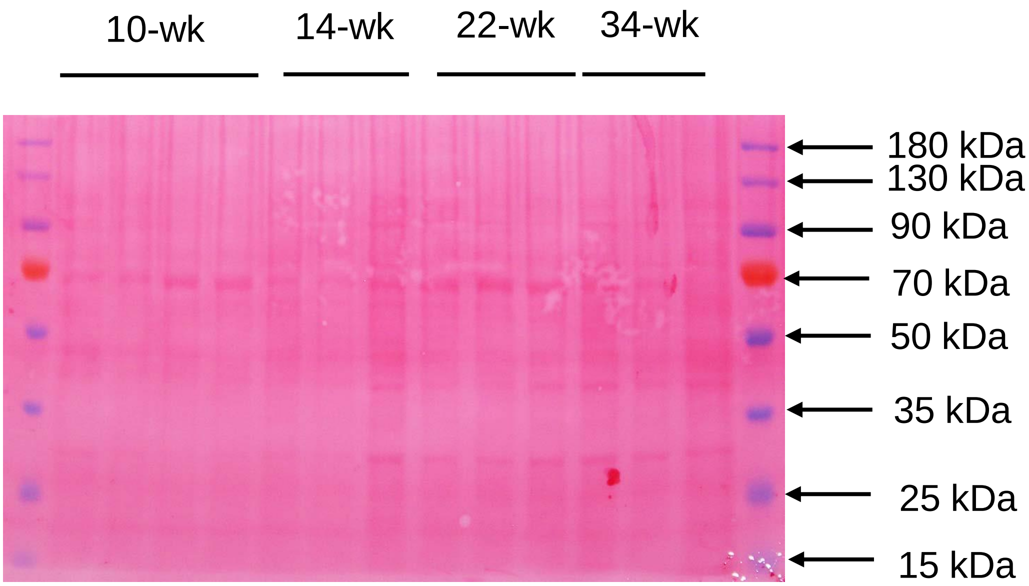

b

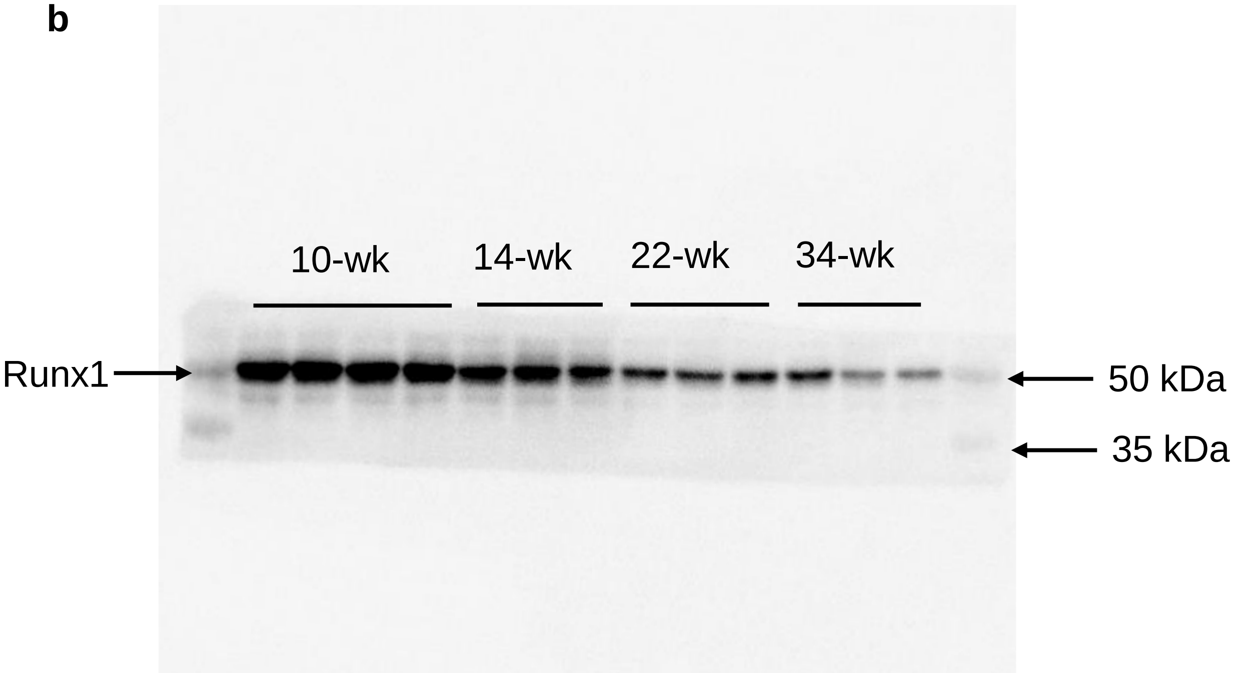

c

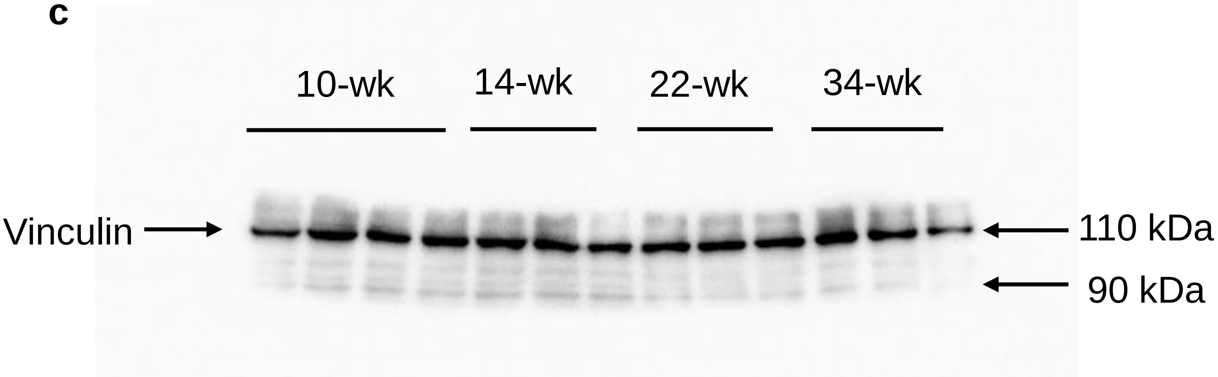

Supplemental Material Figure 1. Original Western blot images for figure 1C. (a) Full-membrane dyed with Ponceaux. (b) Full-length blot section for Runx1. (c) Full-length blot section for Vinculin.
